# Indices of immune function used by ecologists are mostly unaffected by repeated freeze-thaw cycles and methodological deviations

**DOI:** 10.1186/s12983-017-0226-9

**Published:** 2017-09-01

**Authors:** Arne Hegemann, Sara Pardal, Kevin D. Matson

**Affiliations:** 1 Department of Biology, Lund University, Ecology Building, SE-223 62 Lund, Sweden; 2 MARE - Marine and Environmental Sciences Centre, Department of Life Sciences, University of Coimbra, 3000-456 Coimbra, Portugal; 3 Resource Ecology Group, Environmental Sciences Department, Wageningen University, 6700 AA Wageningen, The Netherlands

**Keywords:** Eco-immunology, Immunity assays, Assay methodology, Avian, Pre-analytical error, Sample stability, Repeated defrosting

## Abstract

**Background:**

Over the past couple of decades, measuring immunological parameters has become widespread in studies of ecology and evolution. A combination of different immunological indices is useful for quantifying different parts of the immune system and comprehensively assessing immune function. Running multiple immune assays usually requires samples to be repeatedly thawed and re-frozen. There is some evidence that repeated freezing and thawing can affect assay results, but this has never been comprehensively studied in some common ecological immunology assays. We tested the effect of multiple (1, 2, 3, 4, 5, 10) freeze-thaw cycles on the results of four commonly used immunological assays: haemolysis-haemagglutination titres, haptoglobin concentration, bacterial killing capacity and total immunoglobulins (IgY). We tested five different bird species from four different bird orders (Passeriformes, Columbiformes, Charadriiformes and Galliformes), and we included both captive and free-living individuals. In addition, we tested for haptoglobin concentrations and the haemolysis-haemagglutination assay if re-analysing samples 1 year apart led to different results. For the haemolysis-haemagglutination assay we also tested two different sources of rabbit blood, and we compared untreated microtitre plates with plates that were “blocked” to prevent nonspecific interactions between the plate and assay reagents.

**Results:**

Repeated freezing and thawing of plasma had no effect on lysis titres, haptoglobin concentrations, bacterial killing capacity, or total immunoglobulin levels. Agglutination titres were unaffected by up to five cycles but were lower after ten freeze-thaw cycles. For the haemolysis-haemagglutination assay and haptoglobin concentrations, re-analysing samples 1 year apart yielded highly correlated data. For the haemolysis-haemagglutination assay, the source of rabbit blood did not influence the results, and the untreated vs. blocked plates differed slightly overall, but at the individual level assay results were highly correlated. Using different rabbit blood sources or different types of microtitre plates yielded highly correlated data.

**Conclusions:**

Our data suggest that repeated freeze-thaw cycles do not impair assay results to the point of influencing ecological or evolutionary conclusions. Plasma samples can be safely stored in one tube and thawed repeatedly for different assays. Nevertheless, we recommend consistent treatment of samples in terms of freeze-thaw cycles or other laboratory treatments to minimize the potential for introducing a systematic bias.

## Background

In the mid 1990’s, evolutionary ecologists began to consider that the costs and benefits associated with the immune system might lead to important trade-offs between defences against diseases and other behavioural and physiological processes that impact individual fitness [1–3]. Since then, several techniques to quantify immune function have become available, and measuring different immunological parameters in free-living and captive animals has become widespread in studies of ecology and evolution. In fact, given the complexity of the immune system, a combination of assays targeting different parts of the immune system is favoured in order to gain an understanding that is as comprehensive as possible. Numerous publications have stressed the importance of measuring more than one immune parameter, including parasite-specific assays [4–10].

Although multiple immune assays are potentially available when testing innate and adaptive immune function [4, 9, 11], a few assays to measure baseline immune function are particular widespread in ecological and evolutionary research. The haemolysis-haemagglutination assay (HLHA) allows the quantification of complement (measured as lysis titres) and natural antibodies (measured as agglutination titres) [12]. Other assays measure an acute phase protein (haptoglobin) concentrations [10, 13, 14], bacterial killing capacity [15–17] and total immunoglobulin levels [18]. All of these immunological assays can be done using previously frozen plasma (or serum) samples. This advantage, combined with the assays’ relative simplicity and inter-specific utility, has translated to a widespread application in ecological and evolutionary studies, especially in studies where logistical constraints (often related to the working conditions with free-living animals) pose challenges. In fact, many studies try to combine two or more of these assays to get a more comprehensive view on immune function [19–24].

Running multiple immune assays on the same individual often results in samples that experience multiple freeze-thaw cycles. Freeze-thaw cycles can degrade samples by altering chemical composition and molecular function [25]. For example, in humans and other mammals, some common clinical chemistry analytes are affected by repeated defrosting, while others remain stable even after multiple freeze-thaw cycles [26–28]. This opens the questions whether repeated defrosting also impacts assays used in studies in ecology and evolution. Yet, to the best of our knowledge, data on whether freeze-thaw cycles impair the results of many commonly used assays in ecological immunology are not available. One exception is the assay of bacterial killing capacity in which data suggest that repeated freezing and thawing lead to decreased killing [17].

Here, we present a comprehensive study testing if repeated freeze-thaw cycles influence the results of four immune assays (haemolysis-haemagglutination titres, haptoglobin concentrations, bacteria killing capacity and total immunoglobulin levels) frequently used in studies of ecology and evolution. We used samples from five bird species representing four different orders. Furthermore, we also systematically examined several other methodological concerns. With haemolysis-haemagglutination titres and haptoglobin concentrations, we also explored the relationships between results from duplicate intra-sample analyses when the assays were separated by a year. Lastly, we tested if lysis and agglutination titres depended on the source of rabbit red blood cells (used as an antigen-rich particulate) or on microtitre plate characteristics.

## Methods

### Sample collection and storage

We sampled five different bird species from four different orders: free-living adult and nestling Jackdaws (*Corvus monedula*) from a study colony in Sweden during the 2015 breeding season, adult free-living Common Blackbirds (*Turdus merula*) and Ruffs (*Philomachus pugnax*) from Portugal between February and May 2015, adult Homing pigeons (*Columba livia f. domestica*) from a captive population in The Netherlands in the summer of 2015 [13], and Lohman White breed chickens (*Gallus gallus domesticus*) from a population housed at Lund University in 2015 [29]. We collected blood samples from the brachial vein using heparinised microhaematocrit capillary tubes or syringes (in case of pigeons). Samples were chilled (4 °C for pigeon samples only; on ice all other samples) until centrifugation to separate the cellular and plasma fractions. After centrifugation, several individual samples were pooled (within species and age class) and then divided into a maximum of six different aliquots and frozen. We made several series of up to six different aliquots for the four different immune assays that we tested. For Blackbirds and Ruffs, samples were divided into different aliquots after an initial freeze; hence, the first freeze-thaw cycle (see below) is missing for these species. We pooled three adult Jackdaws and additionally pooled six nestling Jackdaws and ran both pools in triplicate. We pooled five Blackbirds and ran this pool in triplicate. We pooled five Ruffs and ran this pool in duplicate. We ran two pools from five chicken in triplicate. We ran, in duplicate, samples from five individual Pigeons (three males and two females) and two pools (one of five males and one of four females).

### Experimental set-up

Samples were moved from a − 20 °C freezer to a lab bench to thaw at room temperature for 30 min. All samples were completely melted after this period. Samples were then re-frozen at-20 °C degrees for at least 6 h before the next freeze-thaw cycle began. We produced separate aliquots with 1, 2, 3, 4, 5 or 10 freeze-thaw cycles for each of the four immune assays. The samples with only one freeze-thaw cycle were collected, frozen, thawed, and then directly analysed together with all other samples, i.e. the samples undergoing 2, 3, 4, 5 or 10 freeze-thaw cycles. While each freeze-thaw cycle was carried out, any samples assigned for fewer freeze-thaw cycles remained in the freezer. Before the start of each assay all freeze-thaw cycles were removed from the freezer. Hence, the amount of time that samples spent in unfrozen conditions directly before the assays were run, was the same for the full range of freeze-thaw cycles, and the total storage age (in days) is equal for the full range of freeze-thaw cycles within a species. Prior to analyses, all samples were randomized. Samples were run as triplicates (Jackdaws, chicken, Blackbirds) or duplicates (pigeons, Ruffs) within the same assay microtitre plate. As running all assays in triplicate for six freeze-thaw cycles requires a large amount of plasma, we could not run all assays for all species after taking ethical and logistical constraints into account. Hence, for some species, we could only ran a subset of assays.

### Haptoglobin

Haptoglobin is an acute phase protein that is released from the liver during a pathogenic challenge [30]. In Jackdaw, pigeon, chicken and Blackbird samples, we quantified concentrations (mg mL^−1^) of this protein (or a functional equivalent, see [13]) in plasma samples using a commercially available colorimetric assay kit (TP801; Tri-Delta Diagnostics, NJ, USA). This functional assay quantifies the heme-binding capacity of plasma. We followed the ‘manual method’ instructions provided by the kit manufacturer with minor modifications following Matson et al. [13]. We measured absorbance at three wavelengths (405, 450 and 630 nm) prior to the addition of the final reagent that initiated the colour-change reaction. We used the pre-scan at the normal assay wavelength of 630 nm to correct for differences in plasma colour and cloudiness by subtracting pre-scan absorbance values from final absorbance values. We used the 405 and 450 nm pre-scan to statistically analyze and correct for differences in plasma sample redness, an indication of hemolysis, which can affect the assay [13]. As our different aliquots were made of the same pool and hence consisted of similar plasma redness, neither of those two wavelengths (405 and 450 nm) explained any variation, and we do not report on those measurements any further.

### Bacteria killing capacity

In Jackdaw, Blackbird and Ruff samples, we quantified the capacity of plasma to kill *E. coli* using the method described by French and Neuman-Lee [16] and first determined the optimal bacterial concentration for each species. Based on this, we then mixed 3 μl of plasma with 4 μl of 10^5^ *E. coli* solution (ATCC 8739; MicLev) for Blackbirds and Ruffs, and we mixed 2 μl of plasma with 4 μl of 10^6^ *E. coli* solution for Jackdaws. Microtitre plates were incubated at 37 °C for 12 h. We measured bacteria growth at 600 nm using a microplate reader [31]. We afterwards calculated bacteria killing ability following French and Neuman-Lee [16]. We used four negative controls per plate to ensure that there was no contamination.

### Haemolysis-haemagglutination

Using Jackdaw, Ruff, pigeon and chicken samples, we quantified complement (measured as lysis titres) and natural antibodies (measured as agglutination titres) following the method of Matson et al. [12]. In brief, plasma samples were serially diluted in microtitre plates and incubated with a 1% rabbit red blood cell suspension (Harlan Laboratories, United Kingdom). Following incubation, plate images were recorded after 20 min (agglutination) and 90 min (lysis). Because pigeons show weak complement activity, in this species, lysis was scored after 24 h rather 90 min (see [13]). Images of individual samples were randomized and scored at least two times, always blindly with respect to sample identity and freeze-thaw cycle number. Lysis and agglutination were recorded as titres (−log^2^ of the last plasma dilution that shows each reaction).

### Total immunoglobulins

We quantified the total level of antibodies (immunoglobulins IgY; the avian equivalent to mammalian IgG) in plasma by means of an enzyme-linked immunosorbent assay (ELISA) following the protocol described by Sköld-Chiriac et al. [32] that has been applied in several eco-immunological studies [18, 31, 33, 34]. Since this particular protocol is for passerines, we analysed only Jackdaw and Blackbird samples. In brief, microtitre plates were coated with goat-anti-bird immunoglobulin G (IgG; Novus Biologicals, catalog no. NB7226) and blocked with 3% powdered milk PBS/Tween 20. We added 100 μl of plasma diluted 1:300 in 1% powdered milk in PBS/Tween 20. We added 100 μl of rabbit-anti–Red-winged Blackbird IgG (1:1000 dilution in 1% powdered milk in PBS/Tween 20). Afterwards we added 100 μl of peroxidase labelled goat-anti-rabbit antibody (Sigma-Aldrich, catalog no. A6154; 1:2000 dilution in 1% powdered milk in PBS/Tween 20). Lastly, we added 100 μl of ABTS (Sigma-Aldrich, catalogue no. A1888) and peroxidase diluted in citrate buffer. Antibody concentrations are represented as the slopes of colour change of the substrate overtime (measured in 10^−3^ optical density per minute [mOD min^−1^]). Ultimately, antibody levels are calculated as the mean of the duplicates minus the mean of the blanks and are transformed based on the plate standards to correct for variation among plates.

### Other methodological considerations

In addition to analysing the series of freeze-thaw cycles under standard assay conditions with a range of species, we conducted additional tests concerning haptoglobin concentrations and lysis and agglutination titres using surplus plasma collected only from pigeons (i.e., different groups of birds collected at different points in time, compared to the plasma used for the freeze-thaw cycles). First, we quantified haptoglobin concentrations and lysis and agglutination titres in 32 pigeon samples that were collected over the course of 2013. Using the standard procedures, we analysed these samples first in the summer of 2014 and analysed them again in the summer of 2015; samples were stored at −20 °C. Second, we concurrently quantified only agglutination in 61 samples from pigeons (housed at University of Kiel, Germany [35]) using the standard protocol, but with rabbit red blood cells from two different suppliers (Harlan, as above, and Hemostat Laboratories, CA, USA). Third, we also analysed 72 pigeon samples using the standard haemolysis and haemagglutination protocol but with untreated microtitre plates (i.e., standard protocol) and plates that were blocked to prevent nonspecific interactions between the plate and assay reagents. After the blocking procedure, which used a 2% powdered milk solution in PBS for 1 h at room temperature, plates were washed three times with PBS/Tween 20 [36]. The abovementioned methodological considerations were only tested on samples from captive pigeons which could be sampled on multiple occasions and allowed to gather the necessary volumes of plasma samples for the designed assays. Due to plasma limitations and ethical considerations, these assays were not performed on free-living bird species, but restricted to pigeons as a model species.

### Statistical analyses

To test for the effects of freeze-thaw cycles on the immune parameters, we used linear mixed models (function lme of package nlme) [37] in R version 3.2.3. Immune parameters served as dependent variables. We included species, freeze-thaw cycle and the interaction between the two as explanatory variables, and sample identity as a random effect. Assumptions of all models were checked on the residuals of the final model.

For the other methodological analyses, we used paired comparisons to test group differences and correlations to evaluate patterns among individuals. Parametric and non-parametric tests gave qualitatively similar results; here we report the parametric test statistics.

## Results

### Repeated freeze-thawing cycles

**Fig. 1:**
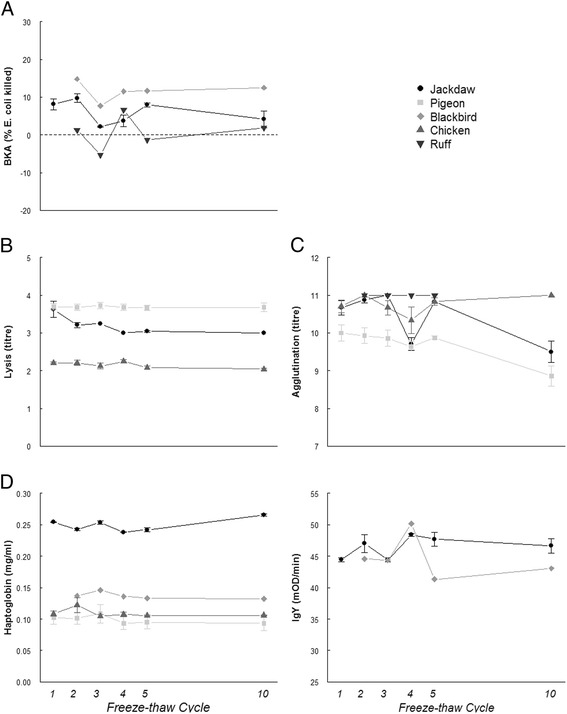
Effects of repeated freeze-thaw cycles on five commonly applied immune parameters in ecology and evolution. **a**) bacteria killing capacity, **b**) lysis titres, **c**) agglutination titres, **d**) haptoglobin concentrations and **e**) immunoglobulin levels. Symbols depict means and standard errors

Repeated freezing and thawing of plasma had no effect on haptoglobin concentrations, lysis titres, bacteria killing capacities or immunoglobulin levels (Table 1; Fig. 1). Agglutination titres declined with increasing freeze-thawing cycles, but this effect was only seen after ten freeze-thaw cycles. When the ten cycles point was excluded, the effect of freeze-thaw cycle on agglutination titres disappeared (F = 1.29, df = 1.45, *p* = 0.262). Species differed in terms of haptoglobin concentrations, lysis titres and agglutination titres (Table 1). However, the interaction between species and freeze-thawing cycle was never significant (Table 1).

**Table 1:** Statistics and coefficients of the linear mixed models of different immune parameters in relation to repeated freeze-thawing cycles

	Haptoglobin conc.	Lysis titre	Agglutination titre	Bacteria killing capacity	Total IgY level
*Freeze-thaw cycle*					
F	1.44	2.94	17.88	0.27	0.04
β	−0.00	−0.01	−0.10	−0.146	0.04
df	1,57	1,56	1,56	1,17	1,13
p	0.235	0.092	**0.001**	0.610	0.842
*Species*					
F	9.89	80.69	5.56	9.95	1.49
df	3,8	3,8	3,8	2,1	1,1
p	**0.005**	**<0.001**	**0.023**	0.219	0.437
Interaction Freeze-thaw cycle * species					
F	4.53	5.73	7.05	1.21	1.29
df	3	3	3	2	1
p	0.210	0.126	0.070	0.546	0.256

*P*-values <0.05 are in bold

### Other methodological considerations

Re-analysis of samples after 1 year of storage resulted in significantly lower haptoglobin (Hp) concentrations (mean Hp in 2014 = 0.127 mg mL^−1^; mean Hp in 2015 = 0.117 mg mL^−1^; *t* = 2.24, df = 31, *p* = 0.033). Across samples, haptoglobin concentrations were correlated between the 2 years (*r* = 0.93, *t* = 14.07, df = 30, *p* < 0.001). Raw absorbance values (i.e., “blueness” measured at 630 nm 5 min after colour change initiation) also showed between-year differences (*t* = −2.50, df = 31, *p* = 0.018) and correlations across samples (*r* = 0.93, *t* = 13.44, df = 30, *p* < 0.001). Re-analysis of samples after 1 year of storage resulted in significantly lower lysis titres (mean lysis for 2014 = 1.8; mean lysis for 2015 = 1.0; *t* = 4.71, df = 31, *p* < 0.001). Across samples, lysis titres were correlated between the 2 years (*r* = 0.78, *t* = 6.89, df = 30, *p* < 0.001). Finally, re-analysis of samples after 1 year of storage lead to similar agglutination titres (mean agglutination for 2014 = 7.5; mean agglutination for 2015 = 7.4; *t* = 0.13, df = 31, *p* = 0.90). Across samples, agglutination titres were not correlated between the 2 years (*r* = 0.15, *t* = 0.80, df = 30, *p* > 0.4). The assay standard (chicken plasma, in duplicate per plate) did not differ between assay years (mean agglutination for 2014 = 5.9; mean agglutination for 2015 = 6.0; t = -1.04, df = 31.061, *p* = 0.31), and within- and among-assay-plate variation was consistently low (average within ≤ 3.9%; among ≤ 5.5%).

The comparison of the two different sources of rabbit blood revealed no effect on agglutination titres (mean agglutination for Harlan = 3.8; mean agglutination for Hemostat = 3.9; *t* = 1.25, df = 60, *p* > 0.2). Across samples, agglutination titres using the two sources were highly correlated (*r* = 0.68, *t* = 7.07, df = 59, *p* < 0.001).

Blocked microtitre plates did not differ significantly from unblocked plates in terms of lysis titres (mean lysis for blocked = 3.5; mean lysis for unblocked = 3.4; *t* = −0.51, df = 71, *p* > 0.6). Across samples, lysis titres arising under the two plate conditions were highly correlated (*r* = 0.86, *t* = 14.12, df = 70, *p* < 0.001). Blocked plates resulted in significantly higher agglutination titres compared to unblocked plates (mean agglutination for blocked = 8.0; mean agglutination for unblocked = 7.5; *t* = −2.51, df = 71, *p* = 0.014). Across samples, agglutination titres arising under the two plate conditions were highly correlated (*r* = 0.60, *t* = 6.24, df = 70, *p* < 0.001).

## Discussion

Measuring immune function in wild animals is constrained by many logistical challenges [5, 7]. Over the last two decades, however, several new assays have become available [11, 12, 16]. Since these assays rely on blood plasma (or serum), which is often collected in small volumes and frozen in microcentrifuge tubes, post-sample collection handling might introduce variation into assay results. We found clear evidence that one such handling concern (repeated freezing and thawing) had virtually no effect on the final outcomes of four assays currently and widely used to study ecological immunology. Only agglutination decreased at the point of ten freeze-thaw cycles. This near absence of effects of repeated freeze-thaw cycles on the results of the immune assays is reassuring for investigators who wish to measure several indices of immune function in single samples from individual animals. Since the stability of samples was consistent across species from four different taxonomic orders and in free-living and captive birds, the resistance to freeze-thaw effect appears to be independent of the species and condition of the sampled individuals. Although we have only tested birds, we can offer no clear reason why results would differ for other vertebrate classes (e.g. Mammalia, Reptilia, etc.), but this remains to be tested. For human samples, repeated freezing to −70 °C and thawing has no meaningful effects on the plasma and serum concentrations of a considerable number of micronutrients and hormones [38].

Our current results are in line with other studies that found no or little effect of multiple freeze-thaw cycles on different chemical analytes of blood [26–28, 38]. However, some studies have suggested freeze-thaw effects, including on parameters we measured here. For example, Boadella & Gortázar [39] advised against carrying out ELISAs with samples with medium to strong haemolysis that have undergone more than three freeze-thaw cycles and with any samples that have undergone more than five cycles. Yet, our study and the study of Pinsky et al. [40] failed to find any effect on the antibody levels of up to ten freeze-thaw cycles. Gutierrez et al. [41] found that haptoglobin concentrations increased significantly by 29% after five freeze-thaw cycles, but in that study, they used a time-resolved immunofluorometric method, in contrast to our functional colorimetric assay which is commonly used in ecological studies. Thus, the different sensitivity to freeze-thaw might result from different methods. Furthermore, different freezer storage temperatures might play an important role on sample degradation and help to explain some of the variation in results among studies. In general, the colder the storage temperature, the smaller the degradation effect [42].

Since storage time in a freezer might also impact the results of immunological assays, we investigated this possibility with haptoglobin concentrations and for lysis and agglutination titres, albeit in a less detailed manner than with our freeze-thaw analyses. Re-analysis of samples after 1 year of storage at −20 °C, generally resulted in values that were significantly correlated with values from the previous year. Such correlations were found for haptoglobin and lysis, but with both of these indices, 1 year of storage resulted in overall declines. In the case of haptoglobin, the between-year differences were not simply a by-product of between-year differences in the standard curves used to calculate concentrations, since raw absorbance values also showed between-year differences and correlations across samples. The correlations we observed suggest that biological patterns at the individual level should be discernible even if samples have aged somewhat. Inclusion of sample age or a factor related to analysis batch in statistical analyses could help to account for the decline due to storage time. However, lab analyses of samples that differ both in age and another factor (e.g. source population identity) that is both of study interest *and* that is confounded with sample age should probably be avoided. In contrast to the other indices, agglutination showed no overall decline due to storage, but across samples, values were uncorrelated between the 2 years. The well-known relative durability of antibodies (compared to, e.g. lytic enzymes) probably underlies the resistance of agglutination to freezing and thawing [43, 44]. The mechanisms underlying agglutination are even resistant to heat treatment designed to inactivate complement in plasma samples [12]. The absence of a correlation between agglutination titres scored from first and second analyses raises questions about the value of this index in studies aimed at explaining variation among individuals. The initial description of this assay focused on differences among groups (species, Matson et al. 2005), and further study of its use at the individual level is needed. However, the assay standard revealed no issues with the assay per se.

Tests aimed at the origin of the rabbit red blood cells and at a characteristic of the assay plates, two other potential methodological sources of variation in the haemolysis-haemagglutination assay, also provided new insights into this assay. The two types of rabbit blood did not differ in agglutination titres (lysis was absent in these assay bouts) and sample values were highly correlated, suggesting that researchers working in different regions of the world and with different accessibility to markets for biological products, should be able to compare assay results, if all else is equal. Assay plates might also differ in terms of non-specific binding depending on manufacturer (Anne Peters, pers. comm.). To systematically gain insight into possible effects of non-specific interactions between antibodies, the red blood cell targets, and the plastic plates, we compared normal plastic assay plates to ones that had been blocked with powdered milk. As would be expected, blocking had no impact on lysis; however, blocked plates had higher agglutination titres on average. Blocked plates probably reduce natural antibodies from binding to plastic, and instead allow these antibodies to bind to the rabbit red blood cells and drive agglutination. Effects of blocking are an unlikely source of variation within an individual study or lab (i.e. all samples should be analysed using the same source of plates, which have been similarly handled). But blocking and potentially other plate characteristics, could influence the results of agglutination and complicate comparisons among studies, particularly if absolute levels (and not intra-study patterns) are being compared.

## Conclusions

To conclude, our data suggest that several commonly used ecoimmunological assays produce consistent results even in the face of several factors that could potentially introduce variation or otherwise influence results. Most notably, repeated freeze-thaw cycles do not impair assay results or study conclusions. Thus, plasma (or serum) samples can be safely stored in one tube and thawed (at least 5–10 times) as needed for different assays. Still, best laboratory practices dictate pre-aliquoting samples per assay whenever logistically feasible. When this measure is not achievable, freeze-thaw cycles, assay order, and other assay parameters should be standardized to the greatest extent possible.
